# Vision-Based On-Site Construction Waste Localization Using Unmanned Aerial Vehicle

**DOI:** 10.3390/s24092816

**Published:** 2024-04-28

**Authors:** Zeli Wang, Xincong Yang, Xianghan Zheng, Heng Li

**Affiliations:** 1 Department of Management Science and Engineering, East China University of Science and Technology, Shanghai 200030, China; wang.zeli@ecust.edu.cn; 2 School of Civil and Environmental Engineering, Harbin Institute of Technology, Shenzhen 518055, China; yangxincong@hit.edu.cn; 3 College of Mathematics and Computer Science, Fuzhou University, Fuzhou 350108, China; 4 Department of Building and Real Estate, The Hong Kong Polytechnic University, Hong Kong 999077, China; bshengli@polyu.edu.hk

**Keywords:** construction waste management, unmanned aerial vehicle, computer vision, long-distance target detection

## Abstract

In the context of construction and demolition waste exacerbating environmental pollution, the lack of recycling technology has hindered the green development of the industry. Previous studies have explored robot-based automated recycling methods, but their efficiency is limited by movement speed and detection range, so there is an urgent need to integrate drones into the recycling field to improve construction waste management efficiency. Preliminary investigations have shown that previous construction waste recognition techniques are ineffective when applied to UAVs and also lack a method to accurately convert waste locations in images to actual coordinates. Therefore, this study proposes a new method for autonomously labeling the location of construction waste using UAVs. Using images captured by UAVs, we compiled an image dataset and proposed a high-precision, long-range construction waste recognition algorithm. In addition, we proposed a method to convert the pixel positions of targets to actual positions. Finally, the study verified the effectiveness of the proposed method through experiments. Experimental results demonstrated that the approach proposed in this study enhanced the discernibility of computer vision algorithms towards small targets and high-frequency details within images. In a construction waste localization task using drones, involving high-resolution image recognition, the accuracy and recall were significantly improved by about 2% at speeds of up to 28 fps. The results of this study can guarantee the efficient application of drones to construction sites.

## 1. Introduction

With the development of human society, more and more construction projects are being carried out in countries all over the world. The total output value of China’s construction industry exceeds USD 3 trillion, while in the United States, this figure exceeds USD 6 trillion [1,2]. While improving the living environment of human beings, it also implies the generation of a large amount of waste. This problem has received extensive attention from researchers [3]. Construction and demolition waste (CDW), including wood, brick, glass, and plastic, is the residue and waste from construction-related production activities [4,5]. The large amount of CDW produced every year results in a significant waste of material and land resources around the world [6,7,8]. Meanwhile, landfilled CDW also generates various forms of pollution [9].

In order to reduce the pollution caused by the construction industry, governments and researchers are committed to promoting the implementation of the 6R strategy, which is recognized as a method that can reduce the pollution of waste, in CDW management through policies. At the same time, recycled construction waste has a high economic value [10]. However, its implementation has not been satisfactory [11]. The outcomes indicate that policies in numerous countries and regions have failed to effectively curb the arbitrary disposal of CDW [12,13,14,15]. This can be attributed to the current reliance on manual labor for on-site CDW recovery, disorganized management practices, and a lack of willingness among builders to collect CDW on site. Consequently, research on automated methods for on-site CDW sorting and collection is imperative to improve the recycling rate and efficiency of on-site CDW management. At present, automation technologies have been widely studied in the field of building construction, including CDW processing, and its effectiveness has been fully proved. Among them, the research on automated CDW processing includes various methods, such as a CDW sorting platform, CDW collection robot, and CDW pile type analysis [16,17,18]. However, whether the construction waste is picked up by mobile robots or collected manually, the huge construction site and the complex road environment hinder the CDW recycling work. Construction waste is always piled up and deposited in various places on the construction site, and the method of searching all the areas by manpower or mobile robots is highly inefficient and time-consuming. Therefore, there is an urgent need for a new method that can quickly and accurately scan an entire construction site and locate all CDW.

**Figure 1:**
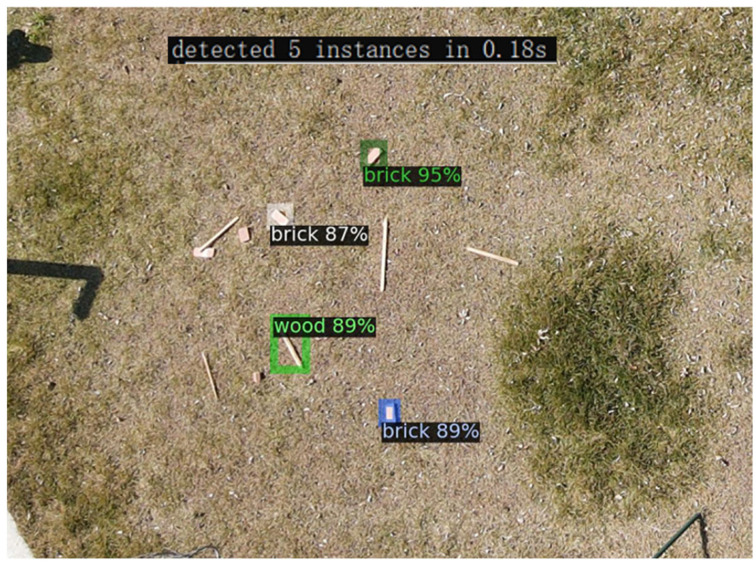
Recognition results and speed.

Unmanned aerial vehicles (UAVs) are currently being used frequently on construction sites [19]. It is faster, more efficient, and less costly compared to mobile robots and humans. Therefore, utilizing UAVs to locate CDW at construction sites can greatly improve the efficiency of construction waste recycling. Preliminary investigations have revealed some challenges in implementing UAV-based automatic target recognition methods at construction sites. On the one hand, experiments have shown that the algorithm used in previous studies yields poor recognition results when processing images taken by drones, with low accuracy and slow speed, as shown in Figure 1. This is due to the small percentage of target pixels in high-resolution images captured by drones, and those algorithms are not sensitive enough to detect these targets. On the other hand, the computer vision algorithms only provide the pixel positions of the targets in the image, and the method of converting these pixel positions into coordinates for use by CDW collection robots and workers is also a problem that needs to be solved.

In order to solve these problems, this study took the most common CDW (brick and wood) as an example and established a 4K high-resolution CDW dataset, which was collected from the perspective of a drone. Next, this study proposed an optimized real-time, small-target recognition algorithm for long-range CDW recognition. Compared with the traditional algorithm, the proposed algorithm can improve the accuracy of small-object recognition while ensuring the recognition speed, helping the UAV achieve fast and accurate real-time detection of CDW on construction sites. Next, this study proposed a coordinate conversion method based on the results of target recognition, so we can automatically mark the location of target CDW. Finally, this study verified the effectiveness of the method through field experiments.

The rest of this paper is organized as follows: Section 2 reviews the state-of-art research on CDW recycling methods and applications of UAV on construction sites. Section 3 presents the design of the proposed method of CDW localization, followed by Section 4, which demonstrates the effectiveness and efficiency of the proposed method. Section 5 concludes this research and discusses the potential of future works.

## 2. Literature Review

### 2.1. Construction Waste Recycling

Although the discussion on the classification and treatment of construction waste has been going on for a long time, in fact, the management of waste in construction projects has not been improved [20]. Construction managers urgently need more new technologies to improve the management level of CDW [21]. Some researchers believe that the government should also invest more funds in new technologies [22]. In CDW management, waste classification is extremely valuable in improving the utilization rate of building materials and the processing efficiency of CDW [23,24]. Therefore, studying how to automatically classify CDW on construction sites is an important path to improve the status quo of construction waste management.

A lot of research has focused on CDW recycling robots, such as the ZenRobotics sorting system developed in 2014 [16]. This system is dedicated to automatically sorting construction waste on the conveyor belt for precise and efficient recycling. Similar research includes the garbage sorting platform developed by Seredkin [25]. The computer vision algorithm proposed by this research can achieve a recognition accuracy of 64%. This kind of CDW sorting platform can help waste treatment plants realize CDW sorting, but it does not help the CDW recycling robot work on the construction sites and cannot fundamentally improve the recycling rate of CDW.

Based on the above reasons, Wang et al. used computer vision methods, such as target recognition and instance segmentation, to analyze the posture of construction waste from the perspective of the robot so as to guide the robotic arm to pick up CDW from the correct angle [17,26]. Although the robot used the optimized path planning algorithm in these studies, it still used a lot of time to search the entire construction site, and the search results also lacked timeliness.

Relevant studies have shown that the use of drones for aerial positioning and commanding unmanned ground vehicles to perform tasks is an inevitable choice for improving the efficiency and effectiveness of automation at construction sites [27]. Therefore, we believe that the introduction of UAVs will greatly improve the recycling efficiency of construction waste on construction sites.

### 2.2. UAV Applications on Construction Sites

Currently, drones have been widely used in construction sites. At the construction site, UAVs can replace workers to realize the remote reading of RFID, significantly enhancing construction management efficiency [28]. UAVs are also utilized in the field of safety management. They are equipped with cameras and scanners to monitor and record crucial areas and combined with 4D BIM technology to achieve efficient construction site safety inspections [29]. In instances of buildings damaged by natural disasters, drones can replace humans to safely extract damage information [30]. However, the tasks in the aforementioned studies are human-controlled, and the current low automation level of UAVs fails to fully utilize their advantages; the accuracy of the results may be disturbed by human error.

UAV application research in construction waste management involves assisting workers in detecting waste around industrial facilities, such as building objects, managing landfills, and estimating waste volumes [31,32,33]. Studies indicate that UAVs have been extensively used in waste management lately, mostly for identifying locations, analyzing spatial characteristics, and assessing environmental safety concerning accumulated waste [34]. Presently, there is limited research on locating CDW dispersed at construction sites.

In conclusion, the current level of UAV-based automation technology used to process on-site CDW is low, with limited related studies. To enhance CDW automatic recycling efficiency on construction sites, research on the UAV-based CDW automatic recycling method, particularly emphasizing developing computer vision algorithms for identifying CDW from UAV perspectives, is essential.

### 2.3. Vison-Based Waste Detection Methods

Over the past decade, computer vision research has steadily increased in fields such as architecture, engineering, construction, and facility management, with applications including performance monitoring, health, safety, and resource management [35,36,37,38]. These studies have made outstanding contributions to the improvement of automation in construction. Among them, many researches on CDW management methods based on computer vision also provide strong technical support for reducing the negative impact of the construction industry on the environment.

Previous studies used GoPro to collect images of construction waste in garbage collection vehicles on construction sites and used image classification algorithms to identify the components of the waste. The study did not consider the mixed pile of construction waste, and it was impossible to accurately identify different construction wastes in the same picture [39]. For CDW mixtures, some studies have proposed a component recognition algorithm based on boundary perception, which aims to automatically analyze the accurate component information of construction waste [40]. Similar effects can also be achieved through semantic segmentation algorithms [18]. This kind of segmentation method based on RGB images has many constraints and cannot guarantee its accuracy in UAV CDW recognition. At the same time, this type of method is limited by the development level of hardware equipment, and it is currently difficult to achieve efficient and real-time target recognition in high-resolution images.

In order to use more information to achieve high-precision recognition, Li et al. collected images and depth information of different CDWs through depth cameras and analyzed them to realize semi-automatic labeling, classification, and contour recognition of various types of construction waste [41]. This algorithm performs well on the sorting platform but is limited by the performance of the depth camera. Therefore, it is difficult to apply this algorithm to outdoor environments, especially long-distance object recognition tasks in UAVs.

Currently, widely used target recognition methods include faster Region-based Convolutional Neural Network (R-CNN), Single-Shot MultiBox Detector (SSD), You Only Look Once (YOLO), etc. [42,43,44]. Among them, YOLO is faster, and the faster R-CNN is more accurate. The YOLO algorithm has been applied in waste recognition, and it has been proven by experiments that the algorithm can achieve an accuracy rate of more than 92% [45]. However, we found that the target object in the dataset established in this study occupies a large number of pixels and has significant edges, which is obviously one of the important guarantees for the good result of the experiment. In the drone’s perspective, the target only occupies less than 5%, and the quality of the edge of the target object cannot be guaranteed, which will greatly reduce the accuracy of the algorithm. Studies have shown that although computer vision algorithms can show good results in some waste identification tasks, they perform very poorly in drones, with only 40.5% accuracy [46]. Therefore, the recognition accuracy of existing computer vision algorithms for small targets are not up to expectations, and optimizing the algorithms for better detection accuracy is one of the current research focuses. For example, Liu et al. optimized the YOLOv3 algorithm to improve its accuracy in the pavement cracks recognition task and finally achieved 87.8% mAP [47].

To sum up, current construction waste recognition algorithms have various defects, especially in the task of long-distance waste recognition. Therefore, it is very important to optimize the long-range CDW recognition algorithm for UAVs, including recognition speed and accuracy.

## 3. Methodology

### 3.1. Data Preparation

**Figure 2:**
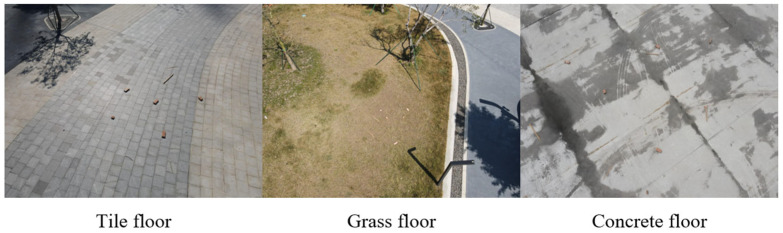
Image dataset.

We collected 1292 images and built a long-distance, high-resolution construction waste dataset, of which 1033 were used as the training set, and the rest were used as the test set. Considering the common types of grounds at the construction site, we collected pictures of wooden strips and bricks in three different backgrounds (tile floor, grass floor, and concrete floor). Some of the data are shown in Figure 2.

Based on the image data, it is evident that the drone-collected 4K images clearly captured the CDW, but the target object occupied less than 5% of the overall image pixels. This implies that the computer vision algorithms spent considerable time processing irrelevant background information during object recognition. Furthermore, if we downsized the image to 41 × 16, as performed in earlier studies, the target features became inconspicuous, leading to a significant reduction in recognition accuracy.

**Figure 3:**
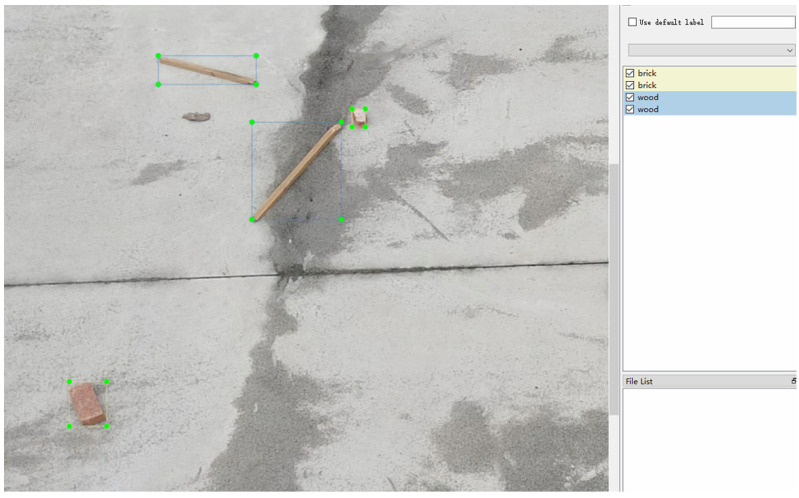
Image data processing (Use the green bounding box to frame the targets).

**Figure 4:**
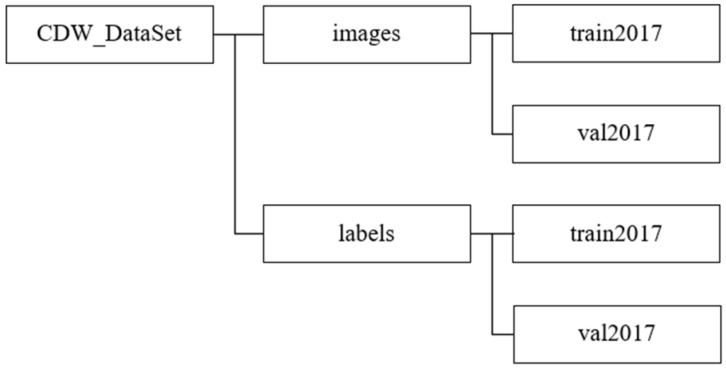
Dataset structure.

For the aforementioned dataset, we utilized LabelImg to process the image data and generate a JSON file [48]. The processing method is shown in Figure 3. Then, we wrote an algorithm to convert the generated annotation files into a standard COCO dataset and finally created a high-resolution COCO format dataset for long-distance construction waste on the construction site [49]. The final dataset structure is shown in Figure 4.

### 3.2. Visual-Based Long-Distance CDW Detection Method

#### 3.2.1. CDW Detection Method

In previous related research, some studies used faster R-CNN as the key technology for CDW recognition. However, experiments and papers show that this method takes about 10 times longer to recognize a single image than the YOLO algorithm [50]. This is not conducive to the rapid search and discovery of CDW by UAVs, especially in the case of high resolution. Therefore, this study used the YOLO series of algorithms as the target recognition method.

**Figure 5:**
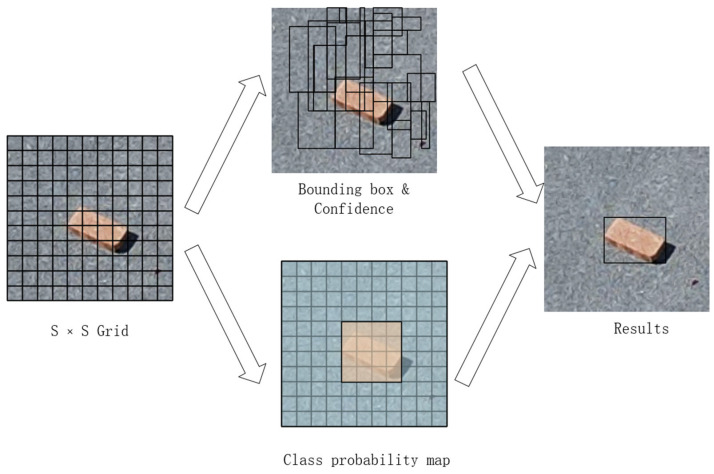
YOLO algorithm flow.

The YOLO algorithm is an end-to-end object detection model that exclusively consists of a CNN (convolutional neural network) model. In the traditional YOLO algorithm, the input image is initially resized to a square (e.g., 44 × 48) and then fed through a CNN network to yield detection results. In the CNN network, the image is divided into multiple grids of size S × S, and each grid is used as the center point of target detection. The bounding boxes and confidence values are also proposed in this step. At the same time, each grid also needs to predict its probability of belonging to a certain class and generate a class probability map. Finally, by combining bounding boxes and class probability maps, the algorithm produces the ultimate object detection results. The specific process is shown in Figure 5.

**Figure 6:**
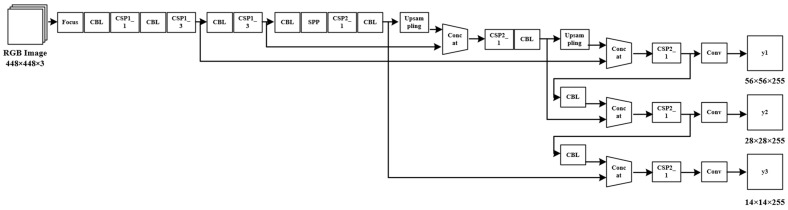
YOLOv5 structure.

YOLOv5 is one of the optimized versions of the YOLO algorithm. YOLOv5 can be divided into four parts: Input, Backbone, Neck and Prediction, as shown in Figure 6. Its innovation includes the following points: First, in the input part, the Mosaic data enhancement method is used to stitch four images using random scaling, random cropping, and random lining up to improve the robustness of the AI model. Second, an adaptive image scaling method is used to automatically scale and fill the image according to the aspect ratio of the input image so that the pixel length values can meet the demand of downsampling 5 times, thus reducing the input size and improving the computational efficiency. Third, the image is processed by using the Focus structure, and the information loss caused by downsampling is reduced in the Backbone part using the operation of intercolumn sampling, splicing, and convolution. Fourth, the design idea of CSPNet is adopted in Backbone and Neck, and two CSP structures are designed to cope with the different demands of different positions in the network.

Preliminary test results showed that the YOLO algorithm can identify the target CDW well at a high resolution, but it cannot guarantee the efficiency of the algorithm. Therefore, we modified the parameters of the image, resizing part of the algorithm to 416 × 416. In this case, the accuracy of the algorithm decreased dramatically, and a large number of targets were missed. Therefore, it was necessary to enhance the sensitivity of the algorithm to small targets, which are expressed as high-frequency information in images.

#### 3.2.2. Optimization

**Figure 7:**
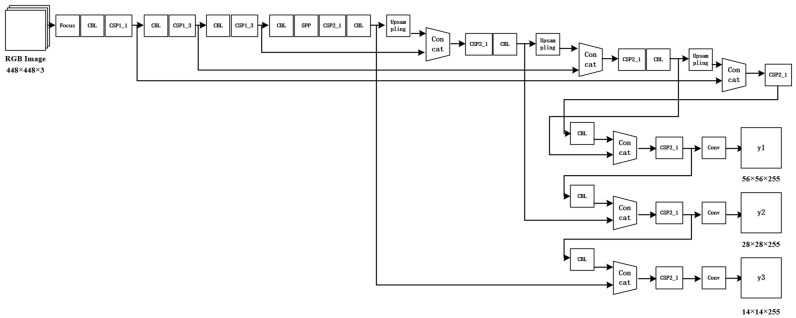
Optimized network structure.

The network structure design plays a crucial role in ensuring the accuracy of computer vision algorithms. While the YOLOv5 algorithm currently available can achieve efficient and precise target recognition, it still falls short in accurately detecting small targets. In a 4K image from the UAV view, the target CDW occupies only a small part of the whole image. Therefore, YOLOv5 compresses the image in the Input section, and the target object occupies only a small number of pixels. Under this premise, multiple downsamplings will result in the features of the target object being difficult to recognize. To address this issue, we optimized the network structure of the YOLOv5 model. We used a short connection structure to obtain features at a lower level. The changes made in the network structure are illustrated in Figure 7, which includes an additional 4-step-long feature map fusion compared to the original YOLOv5 model.

The database is another key to guarantee the accuracy of computer vision algorithms. Therefore, this study enhanced the reliability and usefulness of data through data pre-processing methods. Given that most of the pixels in the 4K images from the UAV viewpoint are invalid information, this study adopted a cropped image approach to enhance the proportion of pixels occupied by the target object in the image. This method can increase the amount of effective information in the training process and improve the training efficiency.

### 3.3. CDW Localization Using an Unmanned Aerial Vehicle

In order to facilitate the calculation, we set the shooting angle of the drone to be vertically downward and set the resolution of the video capture to 4000 × 3000. By checking the equipment parameters, we can easily obtain the horizontal view angle ($\theta_{x}$) and the vertical view angle ($\theta_{y}$). The drone can provide real-time data of the latitude and longitude, orientation (angle to due north $\alpha_{UAV}$), and relative height ($h_{UAV}$). Based on these data and the coordinates of the center point of the target ($x_{t},\;y_{t}$) provided by the computer vision algorithm, we can accurately calculate the latitude and longitude coordinates where the target is located using the following method.

**Figure 8:**
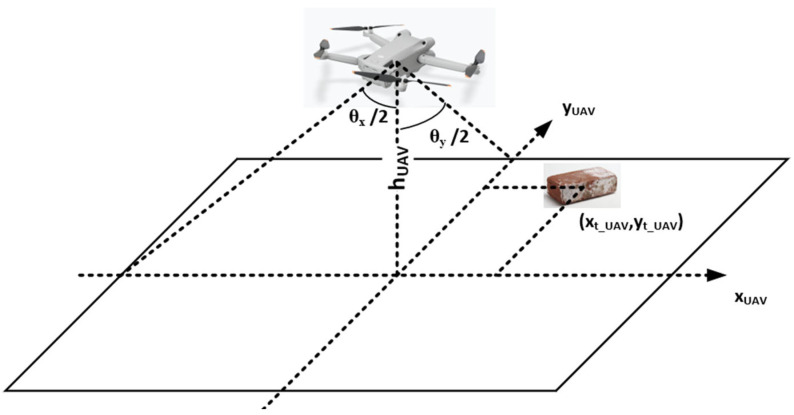
Schematic of the UAV coordinate system.

Step 1: In the acquired image, the projection of the UAV’s position on the ground was located at the center of the image. Therefore, to facilitate the calculation, we established a vertical coordinate system, with the projection of the UAV on the ground as the origin and the UAV facing direction as the *y*-axis, as shown in Figure 8. The coordinates of the target in this coordinate system were $\left(x_{t\_UAV},\;y_{t\_UAV}\right)$, which can be obtained using Equation (1). These coordinates reflect the pixel distance between the target and the UAV projection.

(1) $$\left\{\begin{array}{c} x_{t\_UAV\;}=x_{t}-\frac{4000}{2}\\ y_{t\_UAV}=y_{t}-\frac{3000}{2} \end{array}\right.$$

Step 2: With the relative height of the UAV and the camera view angle data, we can calculate the total distance accommodated by the image captured by the UAV in both the *x*-axis and the *y*-axis directions ($D_{x\_UAV},\;D_{y\_UAV}$), as shown in Equation (2). The actual distance between the target and the UAV in this coordinate system was then calculated and expressed as a vector $\left(D_{t\_UAV\_x},\;D_{t\_UAV\_y}\right)$, as shown in Equation (3).

(2) $$\left\{\begin{array}{c} D_{x\_UAV}=2\times (h_{UAV}\times \tan\frac{\theta_{x}}{2})\\ D_{y\_UAV}=2\times (h_{UAV}\times \tan\frac{\theta_{y}}{2}) \end{array}\right.$$

(3) $$\left\{\begin{array}{c} D_{t\_UAV\_x}=x_{t\_UAV}\times \frac{D_{x\_UAV}}{4000}\\ D_{t\_UAV\_y}=y_{t\_UAV}\times \frac{D_{y\_UAV}}{3000} \end{array}\right.$$

**Figure 9:**
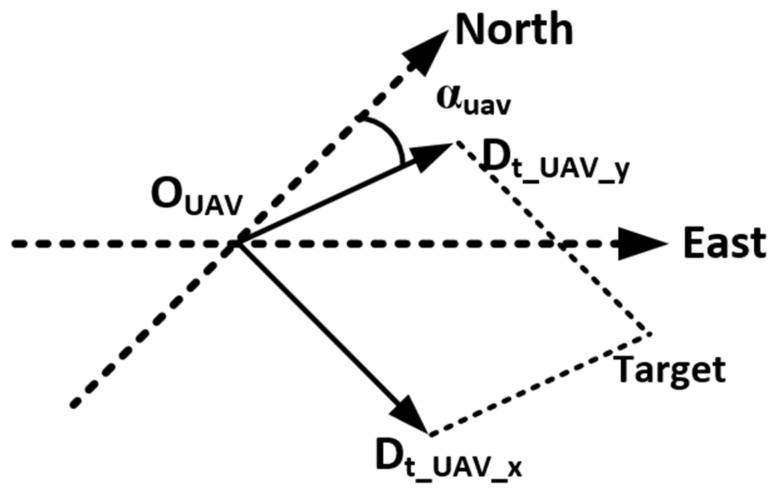
Schematic of the North-East coordinate system.

Step 3: Further, in order to accurately guide the construction waste-picking robot or worker to find the target object, we needed to calculate the specific location of the target object. Therefore, we established a coordinate system, with the projection of the UAV on the ground as the origin and the due north direction as the *y*-axis, as shown in Figure 9. We placed two mutually perpendicular sub-vectors of the previously obtained distance vector in this coordinate system. Equation (4) allowed us to calculate that the target was $D_{t\_E}$ meters due east and $D_{t\_N}$ meters due north of the point where the UAV was located. Further, based on the UAV route and position information, accurate target object localization was able to be obtained.

(4) $$\left\{\begin{array}{c} D_{t\_E}=\sin\left(\frac{\pi }{2}+\alpha_{UAV}\right)\times D_{t\_UAV\_x}+\sin\left(\alpha_{UAV}\right)\times D_{t\_UAV\_y}\\ D_{t\_N}=\cos\left(\frac{\pi }{2}+\alpha_{UAV}\right)\times D_{t\_UAV\_x}+\cos\left(\alpha_{UAV}\right)\times D_{t\_UAV\_y} \end{array}\right.$$

## 4. Evaluations

### 4.1. Experiments and Results

This study employed a server equipped with an i7-11700K CPU and a 3070 GPU, running the Linux 20.04 system based on wsl2 in the Windows 10 environment, as the experimental platform. The system was set up with the CUDA 11.1 and CUDNN 8.4 software, enabling the stable execution of the YOLO algorithms.

The study firstly divided the dataset into an 80% training set and 20% test set randomly; for the key parameters of the model, depth_multiple was set to 0.33, and width_multiple was set to 0.25. Based on this, the head part framework of the YOLOv5n model was optimized according to the method described in Section 3.2.2 to achieve the four-step feature map fusion.

During the training process, we found that the model was able to achieve accurate target object recognition after 600 iterations. In order to accurately compare the recognition ability of different models at different resolutions, we set all training epoch parameters to 600 and tested the YOLOv5n model and our optimized model at 2048 × 2048, 1024 × 1024, 608 × 608, and 416 × 416 resolutions, respectively.

In order to accurately evaluate the effectiveness of the model proposed in this study on the recognition of small targets, the experimental results at each resolution were compared in experiments, mainly comparing the time and accuracy of the different models in recognizing images in the experimental platform. The comparison results are shown in Table 1. For comparison, the experimental results of the faster R-CNN on the same server were 390 ms recognition speed and 94.2% accuracy.

**Figure 10:**
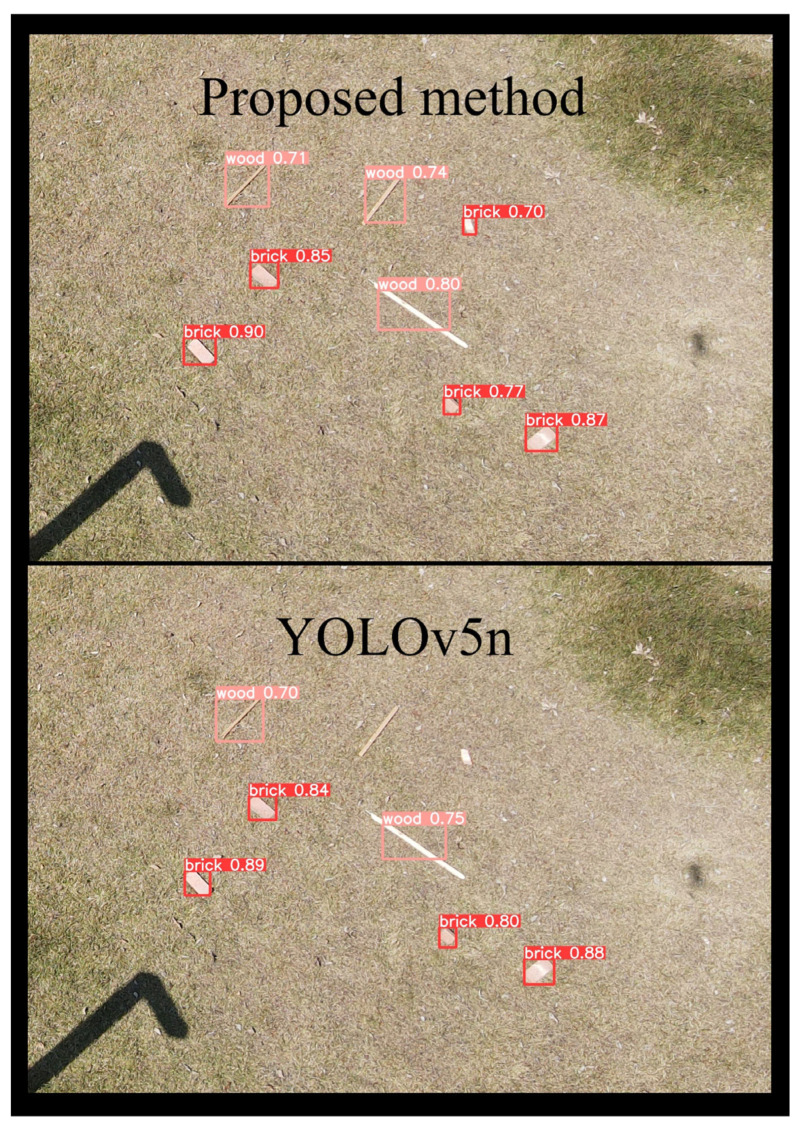
Comparison between YOLOv5n and the proposed method.

The experimental results show that the method proposed in this study is able to slightly improve the accuracy, provided that the image resolution is sufficient. This is due to the target occupying a sufficient number of pixel values, thereby rendering the underlying information inconsequential to the recognition accuracy. However, larger images mean that the model needs to spend more GPU computational resources, which may not only lead to memory overflow, it can also be reflected in the speed of image processing. Experiments show that despite the server support, the recognition speed of large images (2048-pixel and 1024-pixel levels) is still far below the demand for real-time UAV target recognition, and only when the image is reduced to the 416-pixel level resolution can the target recognition speed exceed 25 fps to meet the requirement of real-time CDW recognition. Therefore, the algorithm proposed in this study can improve the accuracy by about 2% when the input image pixel value is set to 416 × 416, which is a significant advantage. The result of the comparative experiment is shown in Figure 10.

**Figure 11:**
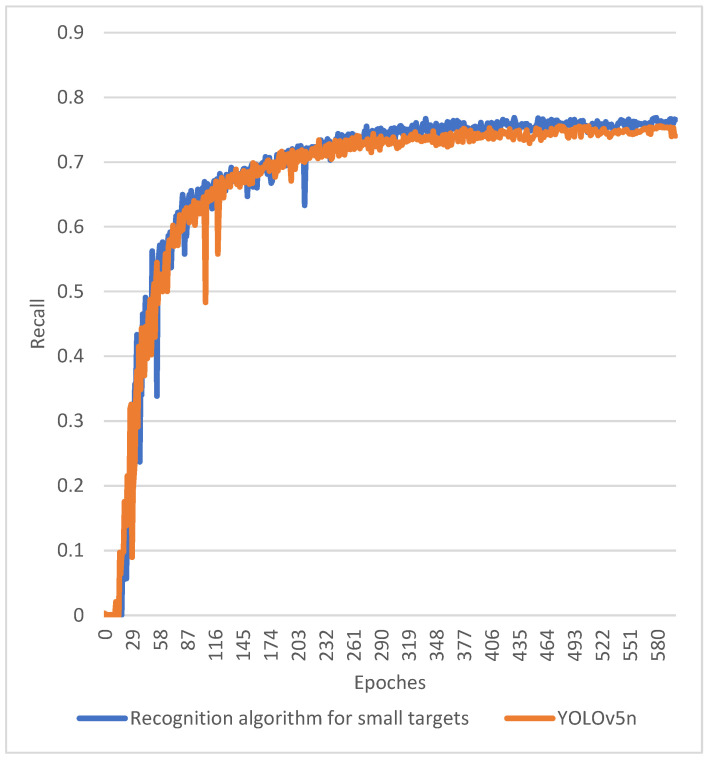
Recall rates for YOLOv5n and the proposed method.

In order to analyze the ability of the algorithms to discover the targets before and after optimization, we compared the training results of the two algorithms by using the recall rate as an analytical metric, as shown in Figure 11. The results showed that the algorithms proposed in this study were able to discover more target CDWs as the number of training times rose.

### 4.2. Discussion

Experiments showed that the algorithm proposed in this study could well achieve the CDW recognition and classification tasks of UAVs at long distances with a maximum accuracy of 98%. In order to meet the need for recognition speed, this study further compressed the image data captured by the UAV to 416 × 416 pixels, which greatly improved the difficulty of CDW recognition. The comparison experiments showed that the algorithm proposed in this study had higher recognition accuracy in the UAV construction waste long-range real-time recognition task, compared with the YOLOv5 algorithm before optimization. The findings of this research provide a novel methodology for detecting CDW distributed across construction sites. Leveraging UAV technology, we addressed the limitations associated with robots’ sluggish mobility and limited detection range. Consequently, the efficiency of construction waste recycling will be significantly improved.

**Figure 12:**
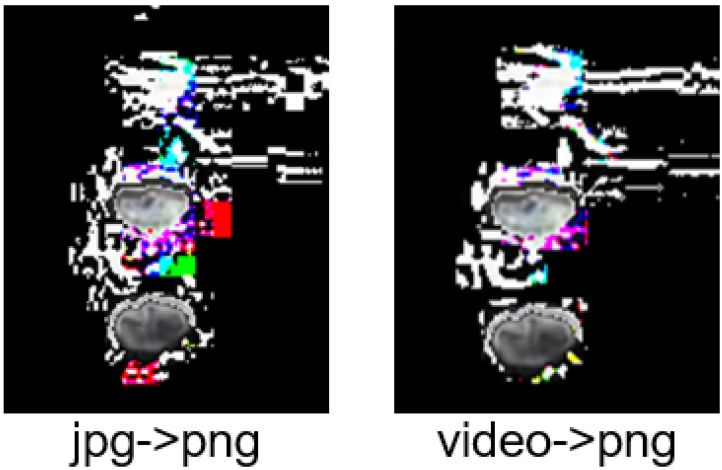
Effect of different format conversion methods on the amount of image noise.

Of course, some shortcomings were found during the experimental process. Firstly, the JPG image format may cause the color distortion of some pixels, which leads to the contamination of the image data and affects the accuracy of target detection. Therefore, in future research, it is necessary to convert the UAV video data directly to PNG format images, which can reduce image noise; a comparison of the two methods is shown in Figure 12. UAV height is also one of the key factors affecting the recognition rate. Being too far away from the target may lead to omission, and this problem exists in all algorithms. In addition, our experiments only selected two kinds of CDWs as target objects. In the future, open target recognition methods can be introduced to improve the generalization ability of the target recognition algorithm without increasing data.

## 5. Conclusions

In this study, we proposed a method to achieve rapid search and localization of CDW at construction sites using UAVs. In order to achieve this goal, a CDW long-range recognition algorithm for UAVs was presented, leveraging prior research. Through the optimization of the YOLOv5 algorithm and the implementation of a four-step feature map fusion method, we enhanced YOLOv5’s recognition accuracy in the CDW dataset collected by drones. We also proposed a coordinate transformation method that converted the recognized pixel position information of a target into real position information that can be easily used by a robot or a worker.

Meanwhile, a long-range construction waste image dataset was collected and constructed using UAV equipment, and the differences in accuracy and recognition speed between the YOLOv5 algorithm and the algorithm proposed in this study under different resolution conditions were trained, tested and compared using this dataset. The study showed that under a 2K resolution, our method can achieve 98% accuracy, which has some advantage over the YOLOv5 algorithm. The working speed of the algorithm at this time did not meet the demand of CDW real-time recognition. Therefore, this study further compared the two algorithms at an image resolution of 416 pixels, and the results showed that our proposed algorithm was able to improve the recognition accuracy by about 2% while ensuring the recognition speed.

In summary, the results of this research can help UAVs to quickly locate on-site CDWs from a long distance, improving the efficiency of CDW discovery at construction sites. Furthermore, it can assist workers or robots to efficiently and accurately recycle construction waste to reduce the environmental pollution problems caused by the construction industry.

Although this study performed well in the evaluation tests, there are still some shortcomings that will be improved in future studies. First, the images of the dataset used JPG format, and the experiment found that some pixel colors were distorted. In future research, we will develop the method of directly converting video data to PNG format image data to reduce the noise of the images so as to improve the recognition accuracy. Secondly, in the future, we will introduce open target detection methods to improve the generalization ability of computer vision algorithms. Finally, in the future, we will conduct application tests at construction sites to verify the effectiveness and application potential of this method.

## Figures and Tables

**Table 1:** Accuracy and recognition speed comparisons.

Image Resolution Method	YOLOv5n		Recognition Algorithm for Small Targets (Proposed Method)	
	Time	Accuracy	Time	Accuracy
2048 × 2048	88 ms	0.968	82.3 ms	0.975
1024 × 1024	72.2 ms	0.948	61.3 ms	0.949
608 × 608	52.8 ms	0.852	48.3 ms	0.870
416 × 416	40.3 ms	0.807	35.6 ms	0.824

## Data Availability

Data are contained within the article.
